# The Secret of the Main Campus Water-Wells, Arba Minch University, Ethiopia

**DOI:** 10.1155/2021/4248505

**Published:** 2021-10-21

**Authors:** Kibru Gedam Berhanu, Asnakew Mulualem Tegegn, Tamru Tesseme Aragaw, Gashaw Sintayehu Angualie, Alemshet Belayneh Yismaw

**Affiliations:** ^1^Arba Minch Water Technology, Department of Water Resources and Irrigation Engineering, Arba Minch, Ethiopia; ^2^Arba Minch Water Technology Institute, Water Supply and Environmental Engineering Department, Arba Minch, Ethiopia; ^3^Woldia University, Water Resources and Irrigation Department, Weldiya, Ethiopia

## Abstract

Groundwater is one of the precious water sources for domestic, irrigation, and industrial demands in arid and semiarid regions of the world. The same is true in Ethiopia context. In this study, seven groundwater samples were collected and analyzed for various chemical constituents (pH, TDS, Na^+^, K^+^, Ca^2+^, Mg^2+^, SO_4_^2−^, Cl^−^, HCO_3_^−^, and NO_3_^−^) to assess the hydrogeochemical characteristics and water types of the groundwater wells. Among the seven sampled groundwater wells, five wells are found and used for domestic water supply in Arba Minch University (AMU) main campus. The remaining two are used for industrial and irrigation demands located at the compound of Textile Factory and Haile Resort, respectively. Results showed that the main campus groundwater wells are saline and harder than the two wells from Textile Factory and Haile Resort. Moreover, elevated concentration of nitrate and potassium (greater than the maximum permissible level allowed in Ethiopia) were obtained in the groundwater sources used mainly in the AMU main campus wells. These elevated concentrations of potassium and nitrate beyond the enriched salt contents in the AMU main campus wells could pose kidney, cardiovascular, and other related health problems. This study, therefore, recommends the AMU to find other groundwater sources for drinking purpose other than the studied water-well field.

## 1. Introduction

Groundwater is a precious resource for our planet, which supports over 97% of accessible freshwater, from which the domestic and irrigation demands cover much [1]. Beyond the quantity, the water quality takes the essential aspect since most of the public health problems are derived from insufficient water supply and sanitation [2]. The poor water quality also has a great effect on soil and crops production, especially in saline-alkali soil areas [3]. Therefore, understanding the quality of available groundwater is necessary for ensuring a reliable supply for domestic, industrial, irrigation, and other purposes. The groundwater quality depends on the physical and chemical characteristics and anthropogenic activities. The physical and chemical factors are owing to the natural factors (comprise of lithology, velocity, geochemical reactions, solubility of salts, and the quality of recharge water) and the human induced anthropogenic activities including agricultural and industrial conditions [4]. Identifying the hydrogeochemical characteristics and groundwater quality is crucial to reveal the interaction mechanism between groundwater and the environment and in turn to provide new insights into water protection and management [5]. The saline groundwater drinking has become an increasing public health especially in the coastal countries of the world [6]. It is identified that a high level of drinking water salinity has posed increased kidney, cardiovascular, and blood pressure [6–8] problems.

Groundwater is at the core of sustainable development and covers more than 70% of the water supply in Ethiopia (IAEA, 2017). The source of water for domestic and recreational (swimming) use at Arba Minch University (AMU) main campus is also groundwater. There are five deep groundwater wells utilized for the intended purpose at this campus. However, the users (including us) of these groundwater wells have been complaining that the water was saline. The prolonged drinking saline water may cause kidney and other health problems for the university students.

Although the health of the whole community of the university, AMU, especially students, has been threatened with drinking water salinity sourced from the water-well fields, the hydrochemical studies were not carried out to date. In this regard, the present study aimed to assess the actual concentration of the main groundwater quality parameters through laboratory analysis to identify which parameters were beyond the limit of Ethiopian Standard Agency and which were not [9], and the reason for health problems. The groundwater quality parameters were analyzed in the laboratory included pH, TDS, Na^+^, K^+^, Ca^2+^, Mg^2+^, SO_4_^2−^, Cl^−^, HCO_3_^−^, NO_3_^−^. The two groundwater samples, which were taken from Arba Minch town, were added in the laboratory analysis just for comparison.

## 2. Materials and Methods

### 2.1. Study Area

**Figure 1:**
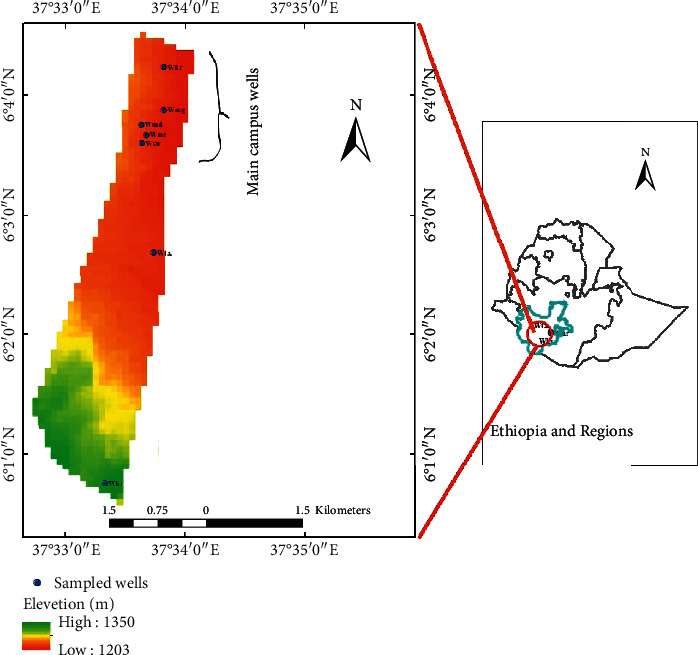
Study area location map.

The study area is AMU main campus (northern part of Figure 1) including parts of Arba Minch town, which is found in Southern Region of Ethiopia. Its geographical location is located approximately between 37^o^32'35” to 37^o^34'0”E and 6^o^0'30” to 6^o^4'35”N (Figure 1). The precipitation pattern of the study area is a bimodal distribution with precipitation peak in April/May and September/October. The amount of rainfall ranges from 62 mm to 162 mm. The intensity of rainfall in the study area varies from year to year and within months of the year. The average maximum temperature of the study area ranges between 28°C and 33.51°C, while the minimum ranges between 15°C and 18°C.

### 2.2. Data Collection

In this particular work, hydrogeochemical analysis of the seven groundwater samples had been carried out at water quality laboratory, AMU. Two groundwater samples were collected from Arba Minch town, one from Haile Resort (labelled as Whr) another from textile factory (labelled as Wtxt). The remaining five samples, namely, Main gate well, Madeya well, Meteorology well, Sewa well, and Kerra well, denoted as Wmg, Wmd, Wmt, Wsw, and Wkr, respectively, were collected from AMU main campus. While these seven groundwater samples were collected, precautions had been made in order to avoid errors during sampling techniques. Among these techniques, all the plastic samplers were appropriately cleansed with distilled water before sample taking and then were transported to the site with icebox. All samples were taken after an average of greater than 30-minute pumping to be sure not to take the pipelines of the well. The containers or samplers were again rinsed with the groundwater well, while after the groundwater samples were taken at each site, the containers of the samples were caped tightly and labelled. Then, the samples were preserved using the icebox and refrigerator until the laboratory hydrogeochemical determination has been completed. Generally, the data collections, the materials used, and the laboratory techniques were summarized in Table 1.

## 3. Results and Discussion

### 3.1. pH Result of the Groundwater Samples (Wells)

**Figure 2:**
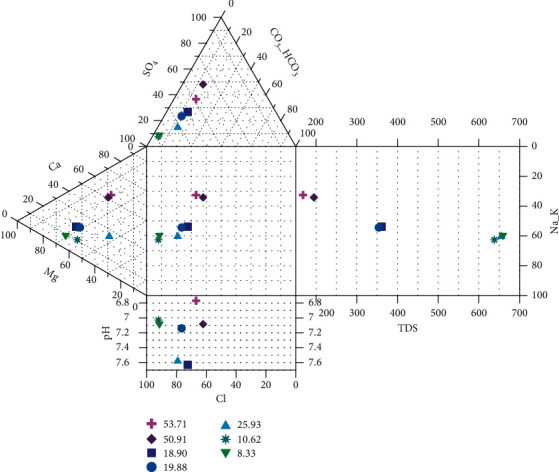
Durov diagram representation of groundwater samples.

The groundwater samples' pH result is tabulated in Table 2 and depicted in Figure 2. The measurement of pH and alkalinity is needed to determine the corrosion of the water. The pH of pure water (H_2_0) is 7 at 25°C, but when exposed to the carbon dioxide in the atmosphere, this equilibrium results in a pH of approximately 5.2. Because of the association of pH with atmospheric gasses and temperature, it is strongly recommended that the water be tested as soon as possible. However, in this research, the in situ pH measurement was not carried out due to scarcity of portable pH meter devices in the laboratory. However, a serious caution was made in order not to expose the samples to atmosphere and light during the sample collection processes.

Water, in general, with a pH < 7, is considered acidic, and with a pH > 7, it is considered basic. The normal range for pH in groundwater systems is 6 to 8.5 as stated by WATER RESEARCH CENTER (WRC) (2021). Although the pH of the water is not a measure of acidic and basic nature and alone does not provide a full picture of the water characteristics, water with a low pH < 6.5 could be acidic, soft, and corrosive. Water with pH less than 6.5 could leach or damage metal ions such as iron, manganese, copper, lead, and zinc from the aquifer, plumbing fixtures and piping [10]. On the other hand, water with a pH > 8.5 could indicate the hard water. Hard water does not pose a health risk but can cause aesthetic problems. These problems include formation of a scale or precipitate on piping and fixtures causing water pressures and interior diameter of piping to decrease.

For the present research, as the laboratory analysis result reveals, the pH values of the sampled groundwater wells were within the range of 6.8 to 7.6, neither acidic nor basic or hard water.

### 3.2. Total Dissolved Solids (TDS), Salinity, and Electrical Conductivity

Total Dissolved Solids (TDS) in groundwater range from 100 to >50,000 mg/l [11]. The laboratory TDS measurement revealed that all sampled groundwater has resulted in 167.8 mg/l to 659 mg/l. The low TDS values were 194.5 and 167.8 mg/l for Wtx and Whr, respectively.

However, the groundwater samples of Wsw, Wmt, Wkr, Wmd, and Wmg revealed elevated TDS values ranging from 361 mg/l to 659 mg/l (Table 3).

Salinity measures the dissolved salts or minerals in water, namely, chloride, sodium, nitrate, calcium, magnesium, bicarbonate, and sulphate. The concentration of boron, bromide, iron, and other trace ions can be locally important [11]. The salinity was measured in per million, which was varying from 0.17 to 0.66 (Table 3). The Kera well (Wkr) and the Maingate well (Wmg) have the same and high salinity. On the other hand, the Textile Factory well (Wtx) and the Haile Resort well (Whr) have less salinity.

Electrical conductivity (EC) is a measure of water capacity to convey electric current due to dissolved ions. The most desirable limit of EC in drinking water is prescribed as 1, 500 *µ*mhos/cm [12]. The EC of the groundwater wells varied from 349 to 1326 *µ*S/cm (Table 3). The main campus groundwater samples (Wmg, Wsw, Wmt, Wkr, and Wmd) are more enriched in salt content than the Wtx and Whr wells.

### 3.3. Hardness

The hardness of water is due to the presence of polyvalent metallic ions, principally Ca^2+^ and Mg^2+^ [13]. Hard water affects both for domestic and industrial usage. For instance, in lather production, the hard water requires considerable amounts of soap before a lather can be produced. Hard water produces also scale in hot water pipes, heater, boilers, and other units, where the temperature of the water is increased significantly. The chemical equation for this process is shown in

(1) $$\begin{array}{c} {\text{Ca}}^{2+}+2{\text{HCO}}^{3-}⟶{\text{CaCO}}_{3}+{\text{CO}}_{2}+{\text{H}}_{2}\text{O} \end{array}$$

Hardness can be expected in regions where large amounts of limestone are found, since water with carbon dioxide will dissolve limestone, releasing the calcium ion. Hardness is measured in terms of CaCO_3_, and the degree of hardness was listed in many books [14, 15] as shown in Table 4.

In general, the hardness of water must be known to determine its use, amount of chemicals required for lime-soda softening, and the design of ion exchange softening units, and the like. Hardness can be determined in the laboratory applying different techniques. In the present case, it was determined using the ethylene diamine tetra acetic acid (EDTA) titration method and applying the following formula:

(2) $$\begin{array}{c} \text{hardness }\left(\text{in }\text{mg}/\text{l}\text{ as }{\text{CaCO}}_{3}\right)=\frac{\left(V\times N\times 50\times 1000\right)}{\text{Sv}}\left(50\right), \end{array}$$

where *V* = volume of titrant (mL); *N* = normality of EDTA; 50 = equivalent weight of CaCO_3_; and Sv = sample volume (mL).

50 ml of groundwater samples was taken for both the total hardness and the calcium hardness determination in 0.01 N of EDTA titration. The magnesium (Mg^2+^) hardness was then found by subtracting the calcium hardness from the total hardness (Table 5). According to Reynolds and Richards [14] classification, the laboratory result revealed that the sampled water wells are grouped into soft, moderately hard, and hard. Wtx and Whr are soft waters, whereas the main campus wells vary from moderately hard to hard waters. Wmg is the hardest one among the main campus wells (Table 5).

### 3.4. Alkalinity

Alkalinity is the measure of the ability of water to neutralize acids. Alkalinity in natural waters includes mainly CO_3_^2−^, HCO_3_^−^ and OH^−^ resulting from the dissolution of mineral substances in the soil and atmosphere. If P is the amount of acid required to reach pH 8.3, and *M* is the total alkalinity of acid required to reach 4.5, the following generalizations can be made to determine the dominant species of the total alkalinity. If *P* = *M*, all alkalinity is OH^−^:

 
*P* = *M*/2, all alkalinity is CO_3_^2−^
 
*P* = 0 (i.e., initial pH is below 8.3), all alkalinity is HCO_3_^−^
 
*P* < *M*/2, predominant species are CO_3_^2−^ and HCO_3_^−^
 
*P* > *M*/2, predominant species are OH^−^ and CO_3_^2-^

The CO_3_^2−^ would then be measured by 2*P* and the HCO_3_^−^ would be measured by the remainder (*M* − 2*P*) [16].

In the present study, the titration technique has been applied to obtain the *P* and *M* values. The final calculation of the alkalinity species is then tabulated in Table 6. As can be depicted in Table 6, only the Wsw and Wkr have both CO_3_^2−^ and HCO_3_^−^ alkalinity constituents, whereas the remaining groundwater samples have only the HCO_3_^−^ dominant alkalinity.

### 3.5. Cations

#### 3.5.1. Sodium Ion (Na^+^)

Sodium ion is omnipresent in water because of the high solubility of many sodium salts. Groundwater typically contains higher concentrations of minerals and salts than do the surface. The children and the elderly are more sensitive than the young to high sodium intake. The increased sensitivity to children is associated with the lower ability of the immature kidney to control sodium levels compared with that of the adult kidney. The elderly have a higher incidence of cardiovascular disease including high blood pressure that makes the elderly more sensitive in high sodium intake than the young [17]. The maximum permissible limit of sodium ion according to Ethiopian standard is 200 mg/l. All the study samples resulted in less than the Ethiopian permissible sodium level. The minimum value was recorded at the textile factory well (Wxt = 15.8 mg/l), and the maximum sodium level was seen at Kera well (Wkr = 165 mg/l), indicating salt enrichment at Wkr.

#### 3.5.2. Potassium Ion (K^+^)

Potassium is an essential element in humans and is rarely found in drinking-water at levels that could be a concern for healthy humans. It can occur in drinking-water as a consequence of the use of potassium permanganate as an oxidant in water treatment. As per ESA [9], the maximum permissible concentration of potassium ion is 1.5 mg/l. All the sampled groundwater wells laboratory analysis revealed the greater values from the maximum permissible level of potassium ion.

The minimum potassium ion measured in the groundwater samples was 1.6 mg/l, whereas the maximum value was 2.4 mg/l (Table 7). This may pose adverse effects on users. Individuals that are most at risk are primarily those in which excretion of potassium ions might be reduced or compromised, including those with kidney disease or renal insufficiency. Older individuals have reduced physiological reserve in their renal function, as well as individuals with other conditions (heart disease, coronary artery disease, hypertension, diabetes, adrenal insufficiency, and existing hyperkalemia). In addition, infants may also be more vulnerable because of a limited renal reserve and immature kidney function [18].

### 3.6. Anions

#### 3.6.1. Chloride (Cl^−^)

Chloride is widely distributed generally in the form of sodium chloride, potassium chloride, and calcium chloride salts. The presence of chlorides in natural waters can be attributed to dissolution of salt deposits, discharges of effluent from chemical industries, seepage discharges, irrigation drainage, contamination from refuse leachates, etc. Each of these sources may result in local contamination of both surface water and ground water. The taste threshold for chloride in drinking water is dependent upon the associated cation but is usually within the range of 200–300 mg of chloride per litre. Taste threshold levels for chloride from sodium chloride, potassium chloride, and calcium chloride in drinking water are 210, 310 and 222 mg/litre, respectively [19].

Chloride concentration was determined through titration technique using the standard silver nitrate titrant 0.141 N and applying the following equation:

(3) $$\begin{array}{c} \text{Cl ion}\left(\frac{\text{mg}}{\text{L}}\left(V1-V2\right)\times N\times 35400\right)=\text{sample volume}\left(\text{ml}\right), \end{array}$$

where *V*1 = volume of titration for sample = 50 ml; *V*2 = volume of titration for blank = 1.2 ml; and *N* = 214 normality of the titrant = 0.0141 N. The calculation was done and tabulated (Table 8).

#### 3.6.2. Nitrate (NO_3_^−^)

Natural nitrate levels in groundwater are generally less than 10 mg/l NO^3−^. But the increasing use of artificial fertilizers, the disposal of wastes (particularly from animal farming), and changes in land use are the main factors responsible for the elevated concentration of nitrates in groundwater supplies. 219 Individual wells in agricultural areas throughout the world especially contribute to nitrate-related toxicity problems, and nitrate levels in the well water often exceed 50 mg/l [20]. The concentration of nitrate in the groundwater wells was determined using the Hach DRTM 2008 spectrophotometer. The concentration level varied from 37.4 to 53.5 mg/l NO_3_^−^ (Table 3), indicated the greater value from the nitrate level available in natural groundwater.

#### 3.6.3. Sulphate (SO_4_^2−^)

**Figure 3:**
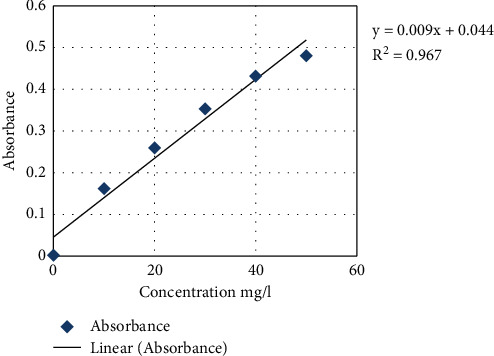
Standard sulphate concentration graph.

The absorbance values of sulphate in sampled groundwater wells were analyzed using UV-5100 spectrophotometer at 420 nm, and the results are presented in Table 9, from which the concentration of sulphate was calculated by using the standard sulphate graph (Figure 3). The results indicated that the maximum value of 31 mg/L of sulphate was observed at Wmd and the lower value of 18.444 mg/l at Wtx. WHO [20] recommended that the maximum permitted level of sulphate in water is 500 mg/L. As a safety measure, water with a sulphate level exceeding 400 ppm should not be used in the preparation of baby food. Sulphate gives a bitter or medicinal taste to water if it exceeds a concentration of 250 mg/L. This may make it unpleasant to drink the water. USEPA advisory recommends that the reduced form of sulphate concentrations in drinking water should be either equal to or below 250 mg/l [21]. However, the concentration of sulphate in all the tested samples agrees with below the permissible limit as per WHO [20] and USEPA [21]. Equation (4) was used to compute the sulphate absorbance of each groundwater sample as tabulated in Table 9.

(4) $$\begin{array}{c} y=0.009x+0.044, \end{array}$$

where *y* = absorbance and *x* = sulphate concentration.

Generally, the anions and cations including TDS and pH analyzed in the study area with the maximum permissible limit according to Ethiopian standard agency [9] have been summarized in Table 2.

### 3.7. Piper Diagram Presentation

The piper diagram was used in the present study since it is the most useful diagram for representing and comparing water quality. All ions except the nitrate ion, which were found in all groundwater samples using different laboratory techniques, were converted to mill equivalent percentage (Table 10). The piper diagram presentation of the chemical analysis was done using the Grapher version 15.

**Figure 4:**
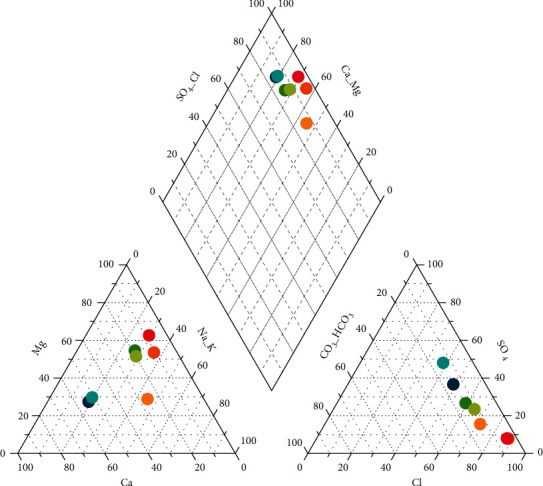
Piper diagram representation of the hydrogeochemical analysis and water types.

The similarities and differences among water samples were presented using the diagram in Figure 4.

As can be understood from Figure 4, the laboratory results fell under the category of Ca-SO_4_ type, Mg-dominant Ca-Mg-Cl type, and Ca-dominant Ca-Na–Cl type. Among the main campus wells, Kera groundwater well (Wkr) is Ca dominant Na- Cl type indicating saline and deep ancient groundwater. The remaining wells (Wsw, Wmt, Wmd, and Wmg) are the Mg dominant Ca-Mg- Cl type, signifying mineral dissolution and interaction between rock and water and the secondary saline water [22, 23]. On the other hand, the groundwater wells, which are out of the main campus (Wtx and Whr), are grouped under the water type of Ca- SO_4_ revealing the typical of gypsum and mine drainage. Further elucidation can also be presented using the Durov Diagram (Figure 2).

The groundwater samples plot appeared closer to the Cl field and far away from the CO_3_ and HCO_3_ fields with their corresponding TDS (200–650 mg/l) and pH values (6.8–7.6) (Figure 2), indicating that these waters interact with the rock matrix and the surrounding environment [24].

## 4. Conclusion

As per the results of the present study, the following conclusions were drawn. The AMU main campus groundwater wells are harder than the sampled groundwater wells at Arba Minch town. But all the sampled groundwater sources are under maximum permissible hardness (300 mg/l) as per the Ethiopian standard agency. The water types of the AMU main campus groundwater wells differ from the groundwater wells located at Arba Minch town. The four groundwater wells of AMU main campus (Wsw, Wmg, Wsw, and Wmt) are of mainly (Ca-Mg-Cl) type of waters. The remaining groundwater well (Wkr) is Ca-Na-Cl water type. These are more saline waters than those of Arba Minch groundwater wells (Wtx and Whr). The Wtx and Whr are of Ca-SO_4_ water type, indicating the typical gypsum derived aquifer. High Ca content, greater than the maximum permissible limit, has been observed at Wkr, whereas the remaining groundwater wells are within the permissible limit. Unlike the two Arba Minch town groundwater wells, highly deviated Mg content from the permissible limit was obtained in all the main campus wells.

All groundwater samples have higher potassium content much greater than the permissible limit of the country. We speculated that the high potassium content, besides the enriched salt content, was the secret why the AMU main campus water-wells have been suspected to cause kidney illness and other health problems. The total hardness, the total alkalinity, the sulphate, and pH of all the samples are within the maximum permissible limit. The nitrate content of Wtx and Whr revealed no greater value from the permitted limit; however, Wsw, Wkr, and Wmg showed greater values. Generally, the AMU main campus groundwater wells have higher chemical contents than the two groundwater wells at Arba Minch town. In general, we posit that the five wells found in AMU main campus should not be used as drinking water because of health threats.

## Figures and Tables

**Table 1:** Water samples, materials used to sample, and laboratory techniques.

Gw samples	Materials used to sample	Laboratory techniques/apparatus	Purpose
7 Gw samples labelled as (i) Wtx (ii) Whr (iii) Wm (iv) Wmd (v) Wmt (vi) Wsw and (vii) Wkr	(i) 2 polypropylene bottles (ii) Icebox and refrigerator to preserve samples (iii) GPS to record the geographic coordinate	EDTA titration	For total hardness and calcium hardness measurements
		Silver nitrate titration	For chloride concentration measurement
		UV.VIS. spectrophotometer	To read the sulphate absorbance of samples
		Hach DR 2800^TM^ spectrophotometer	For nitrate measurement
		Model 2655–10 dual-channel flame photometer	To measure directly the K^+^ and Na^+^ concentration

**Table 2:** Hydrogeochemical analysis result and maximum permissible limit as per ESA, 2013.

Well ID	Ca (mg/l)	Mg (mg/l)	Na (mg/l)	K (mg/l)	Cl (mg/l)	SO_4_ (mg/l)	NO_3_ (mg/l)	TA (mg/l)	TH (mg/l)	TDS (mg/l)	pH
Wtx	42.0	13.0	15.8	1.9	18.0	18.4	41.4	9.5	55.0	167.8	6.8
Whr	48.0	17.0	19.5	2.4	16.0	27.3	37.4	10.0	65.0	194.5	7.1
Wsw	40.0	70.0	63.5	1.6	44.9	27.4	51.5	16.7	110.0	361.0	7.6
Wmt	42.0	66.0	68.2	1.8	52.9	25.9	40.8	16.5	108.0	354.0	7.1
Wkr	83.0	56.0	165.0	1.7	95.8	28.2	51.8	28.4	139.0	655.0	7.6
Wmd	35.0	107.0	134.4	1.9	257.6	31.0	43.7	20.0	142.0	638.0	7.0
Wmg	36.0	164.0	142.9	1.9	244.6	30.2	53.5	22.0	200.0	659.0	7.1
ESA maxpl (mg/l)	75	50	200	1.5	250	250	50	200	300	1000	6.5–8.5

*Note.* TA = total alkalinity, TH = total hardness, and ESA maxpl = Ethiopian Standard Agency maximum permissible limit.

**Table 3:** Concentration of TDS, EC, and salinity including nitrate.

S. no.	Parameters	Well name	Wtx	Whr	Wsw	Wmt	Wkr	Wmd	Wmg
1	EC (microSeimen/cm)		349	404	738	726	1318	1285	1326
2	TDS (mg/l)		167.8	194.5	361	354	655	638	659
3	Salinity per mill		0.17	0.19	0.35	0.35	0.66	0.64	0.66
4	NO_3_^−^ (mg/l)		41.4	37.4	51.5	40.8	51.8	43.7	53.5

**Table 4:** Degree of hardness [15].

Hardness, mg/l as CaCO_3_	Degree of hardness
1–75	Soft
75–150	Moderately hard
150–300	Hard
300 and more	Very hard

**Table 5:** Ca^2+^ and Mg^2+^ hardness determination.

Wells	V (ml)	NEDTA	Eqw CaCO_3_	Factor	Sv (ml)	Th (mg/l)	N EDTA	V (ml)	Ca^2+^ (mg/l)	Mg^2+^ (mg/l)
Wtx	5.5	0.01	50	1000	50	55	0.01	4.2	42	13
Whr	6.5	0.01	50	1000	50	65	0.01	4.8	48	17
Wsw	11	0.01	50	1000	50	110	0.01	4	40	70
Wmt	10.8	0.01	50	1000	50	108	0.01	4.2	42	66
Wkr	13.9	0.01	50	1000	50	139	0.01	8.3	83	56
Wmd	14.2	0.01	50	1000	50	142	0.01	3.5	35	107
Wmg	20	0.01	50	1000	50	200	0.01	3.6	36	164

*Note.* Eqw = equivalent weight; Th = total hardness.

**Table 6:** Determination of alkalinity species.

Wells	P (mg/l)	M (mg/l)	OH (mg/l)	CO_3_^2−^ (mg/l)	HCO_3_^−^ (mg/l)
Wtx	0.000	9.500	0	0	9.500
Whr	0.000	10.000	0	0	10.000
Wsw	0.900	16.700	0	1.8	14.900
Wmt	0.000	16.500	0	0	16.500
Wkr	0.900	28.400	0	1.8	26.600
Wmd	0.000	20.000	0	0	20.000
Wmg	0.000	22.000	0	0	22.000

**Table 7:** Na^+^ and K^+^ concentration levels.

Cations	Wtx	Whr	Wsw	Wmt	Wkr	Wmd	Wmg
Na^+^ (mg/l)	15.8	19.5	63.5	68.2	165	134.4	142.9
K^+^ (mg/l)	1.9	2.4	1.6	1.8	1.7	1.9	1.9

**Table 8:** Cl^−^ concentration levels.

Anion	Wtx	Whr	Wsw	Wmt	Wkr	Wmd	Wmg
Cl^−^ titration with silver nitrate (ml)	3	2.8	5.7	6.5	10.8	27	25.7
V2	1.2	1.2	1.2	1.2	1.2	1.2	1.2
N	0.0141	0.0141	0.0141	0.0141	0.0141	0.0141	0.0141
Sample (ml)	50	50	50	50	50	50	50
Cl^−^ concentration (mg/l)	18.0	16.0	44.9	52.9	95.8	257.6	244.6

**Table 9:** Sulphate concentration from the standard graph line and the absorbance value.

Sampled wells	Absorbance (420 nm)	Concentration (SO_4_^2−^ mg/L)
Wtx	0.210	18.444
Whr	0.290	27.333
Wsw	0.291	27.444
Wmt	0.277	25.889
Wkr	0.298	28.222
Wmd	0.323	31.000
Wmg	0.316	30.222

**Table 10:** Cations and anions equivalent weight in percent.

Well ID	Ca^2+^ (meq%)	Mg^2+^ (meq %)	Na^+^ + K^+^ (meq%)	Cl^−^ (meq%)	SO4^2−^ (meq %)	HCO_3_^−^ + CO_3_^2−^ (meq%)
Wtx	53.7	27.4	18.9	48.5	36.6	14.9
Whr	50.9	29.8	19.3	38.1	48.1	13.9
Wsw	18.9	54.6	26.5	59.1	26.7	14.2
Wmt	19.9	51.5	28.6	64.8	23.5	11.8
Wkr	25.9	28.9	45.2	71.4	15.5	13.1
Wmd	10.6	53.6	35.8	88.2	7.8	4.0
Wmg	8.3	62.6	29.0	87.4	8.0	4.6

## Data Availability

All the required data are available in this article.
